# Trend and outcome of notified children with tuberculosis during 2011-2015 in Kampala, Uganda

**DOI:** 10.1186/s12889-017-4988-y

**Published:** 2017-12-19

**Authors:** Eric Wobudeya, Moorine Sekadde-Kasirye, Derrick Kimuli, Frank Mugabe, Deus Lukoye

**Affiliations:** 1 Mulago National Referral Hospital, Directorate of Paediatrics & Child health, Kampala, Uganda; 2 TRACK TB Project, Management Sciences for Health, P. O. Box, 71419 Kampala, Uganda; 3 Ministry of Health, Uganda National TB & Leprosy Program, P. O. Box, 7272 Kampala, Uganda

## Abstract

**Background:**

The road map for childhood tuberculosis launched in 2013 provided strong renewed efforts focused towards zero deaths due to tuberculosis in children. From 2010, there were efforts to improve childhood tuberculosis diagnosis in Kampala and this study aimed to document the trend and outcome of tuberculosis in children over the period.

**Methods:**

This was a retrospective study of tuberculosis data for Kampala city for the period 2011–2015. We extracted data from the unit TB registers in the 52 Diagnostic and treatment units (DTUs) in the Kampala. We report on data for children 0 to 14 years.

**Results:**

We accessed 33,221 TB patient records of which 2333 (7.0% 95% CI 6.7 to 7.3) were children. The proportion of children with pulmonary TB was 80% (1870/2333) (95% CI 76.7 to 83.7 and extra-pulmonary TB accounted for 20% (463/2333) (CI 18.3 to 21.5). Among pulmonary TB cases, the clinically diagnosed were 82% (1530/1870) (95% CI 80.0 to 83.5) while the bacteriologically confirmed were 18% (340/1870) (95% CI 16.5 to 20.0). Among the bacteriologically confirmed, 45% (154/340) (95% CI 40.1 to 50.6) were smear positive. During the study period 2011 through 2015, the childhood TB notification rate declined as follows; 105, 76, 72, 88, and 74 per 100,000 respectively. The treatment success rate increased from 78% in 2011 to 83% in 2015.

**Conclusions:**

The TB notification rate among children in Kampala city showed a large decline during the period 2011 to 2015. There was a slight improvement in the treatment success rate among the children.

## Background

The Millennium Development Goal (MDG) VI set in 2000 targeted to halve the global tuberculosis (TB) prevalence of 331/100,000 in 1990 by 2015 [1]. The World Health Organization (WHO) global TB reports since 1990 have referenced some of these bench marks to assess progress. The global TB incidence rate declined by 2% each year between 2000 and 2016. The better reporting of cases mainly in India largely explains the increase in notification rates since 2013 [2]. Although the decline in TB deaths by 24% between 2000 and 2016 is significant, it remains the ninth cause of mortality worldwide [2]. The TB associated deaths among HIV negatives reduced from 1.7 Million in 2000 to 1.3 Million in 2016 [2].

The proportion of children notified with TB has progressively increased from 6% in 2012 to the current 10% of the total in 2016 [2, 3]. Reports have also shown a drop in global treatment success rate from 87% in 2013 to 83% in the 2015 cohort largely credited to the large number of “not evaluated” patients [2, 4]. There were 110,000 TB deaths among the HIV negative children, representing 8.5% of all TB deaths in 2016 [2] . Despite the WHO recommendation to start all child TB contacts on isoniazid preventive therapy (IPT), only 13% of child contacts received IPT in 2016 [2]. Reports have shown a decline in childhood notification rates on the background of doubt about the true burden of TB in children with many cases underdiagnosed or under reported. The road map for childhood TB launched in 2013 provided strong renewed efforts focused towards zero deaths due to TB in the <15 years age group [5].

In Uganda, the TB notification rate declined from 209 per 100,000 in 2010 to 161 per 100,000 in 2014 [6]. The treatment success rate improved from 67% in 2009 to 75% in 2013 among new and relapse cases [6–8]. The estimated case detection rate improved from 61% in 2010 to 72% in 2014 [6–8]. HIV testing among diagnosed TB patients improved from 81% in 2010 to 95% in 2014 [6–8], antiretroviral therapy (ART) coverage from 24% in 2010 to 81% in 2014 [6]. Cotrimoxazole preventive therapy (CPT) coverage improved from 90% in 2010 to 98% in 2014 [7, 8]. Data on TB contact investigation in Uganda at the writing of this paper was not available while IPT rolled out at the beginning of 2015. Uganda is one of the countries that achieved a remarkable decline in the prevalence of TB and met the MDG target [9].

Data reported in Uganda so far does not show trend of childhood TB to track progress of TB control in this age group. Projections for childhood TB progress in Uganda are on the background of under reporting and underdiagnosis of TB in children. Over the years, there has been a steady shift from reporting only smear positive cases to all cases including the clinically diagnosed potentially leading to an increase in the reported child TB cases. Even with these improved reporting strategies, the available evidence suggests the true burden of TB disease in children is an underestimate [10]. This reflects the implementation challenges in the diagnosis and reporting of TB in children by the National TB & Leprosy program (NTLP). We showed in our previous report that 7% of the reported TB in 2009 and 2010 in Kampala city were in children [11]. The WHO report covering the same period reported children TB cases represented 1.5% of all smear positive TB cases reported in Uganda [12]. The proportion increased to 7.5% of all the TB cases in Uganda among children in 2013 [6].

From 2013, the NTLP heightened efforts towards improvement of childhood TB diagnosis in Uganda. Such efforts included appointment of a national pediatric TB focal person at the NTLP and mentorships in childhood TB care, recording and reporting. The objective of these interventions was to improve case notification and outcomes of childhood TB. This study therefore aimed to document the trend and describe outcome of child TB cases reported in Kampala city from 2011 to 2015.

## Methods

### Study design

This was a retrospective study of TB records for Kampala city for the period 2011–2015.

### Study setting and data generation

We conducted this study in Kampala city, Uganda’s capital city. Kampala has 5 administrative divisions (Central, Nakawa, Kawempe, Rubaga, and Makindye). Wakiso district, from where much of the Kampala city day population lives, surrounds the city. Children below 15 years in Kampala city for 2011, 2012, 2013 and 2015 were 478,075, 487,830, 497,785 and 518,307 respectively using a baseline population of 507, 942 reported in the 2014 census [13]. We used the simple share population projection formula; baseline population x EXP (growth rate x t) where t is projection time in years from the baseline time point. The census of 2014 showed 2.02% Kampala population increase each year between 2002 and 2014 [13]. The population growth rate for each division is difficult to estimate since in-migrations are not uniform. For this study, we assumed a uniform increase of 2.02% for each division.

This report covers data collected from all the 52 TB diagnostic and treatment units (DTUs) in the Kampala city. Each of the DTUs records TB cases in the Unit TB registers. Health workers complete the unit TB registers at the start of treatment. All the TB medicines are free and testing for HIV is part of the TB and HIV integrated services. The division supervisors transfer information from the Unit TB registers into the division TB registers (both paper and electronic) monthly. The division supervisor files quarterly reports by the 7th of the following month after the end of the quarter to the Kampala city health office for onward transmission to the NTLP. A TB case is a patient in whom a clinician has made a diagnosis of TB either as bacteriologically (smear or Xpert® MTB/RIF or culture positive) or clinically [14].

We included all records of children aged less than 15 years reported with TB in Kampala for the period 2011 to 2015 in the analysis. The data extracted included; age, sex, address, HIV status, ART uptake, CPT uptake, TB classification and outcome.

### Data management and statistical analysis

We extracted data from an electronic TB database. We used Stata transfer v9 to export the relevant extraction from the electronic data to a Stata format and analyzed using STATA v12. We cleaned, coded and explored all the eligible captured records and used descriptive statistics to explore the study population characteristics such as sex, age, HIV status, ART, TB classification, and outcome. Treatment success rate was the proportion of cured plus completed among all childhood TB notifications. We performed exploratory analysis to recognize trends and outcomes of TB in children. We used Chi square for trend test to assess the trend of the reported proportions.

## Results

### Descriptive statistics

We accessed 33,221 patient TB records of which 2333 (7.0% (CI 6.7 to 7.3)) were for children. Of the 2333 children, 53% were males. The median age was 4 years (IQR 9 i.e. 1, 10). Children 0 to 4 years contributed 53% (1241/2333). The proportion with PTB and EPTB among all cases was 80% (1870/2333) (95% CI 78.1 to 81.7) and 20% (463/2333) (95% CI 18.3 to 22.0) respectively. Among pulmonary TB cases the clinically diagnosed were 82% (1530/1870) (95% CI 81.9 to 85.6). The bacteriologically confirmed were 18% (340/1870) (95% CI 14.4 to 18.1) of whom 45% (154/340) (95% CI 41.6 to 54.4) were smear positive. Only 8.7% (162/1870) (95% CI 7.5 to 10.0) of PTB cases had a baseline smear microscopy.

### Trend of TB in children over the study period

There was a general increase in the total TB notifications for all ages but a relative decline in the proportion of children with TB. See other details in the Table 1.

**Table 1:** Trend of TB notifications in children in Kampala district divisions 2011–2015

Age group	Division		2011	2012	2013	2014	2015	*p* value for trend
Overall (adults and children)	Central		626	256	597	1128	1199	
	Kawempe		4054	2440	2866	3475	3040	
	Nakawa		878	457	753	1000	1151	
	Rubaga		1339	533	901	1048	1152	
	Makindye		979	443	788	1448	670	
Proportion of children			7.2%	10%	6.9%	6.3%	6%	0.35
0–4 years	Central	notified	0	0	0	20	18	
		rate^b^	0	0	0	232	204	0.17
	Kawempe	notified	194	147	130	191	117	
		rate^b^	410	304	264	380	228	0.0003^***^
	Nakawa	notified	0	0	4	7	21	
		rate^b^	0	0	9	17	50	0.004^**^
	Lubaga	notified	86	47	50	36	36	
		rate^b^	155	83	86	61	60	<0.0001^****^
	Makindye	notified	33	37	25	27	15	
		rate^b^	64	70	46	49	26	0.0002^***^
5–14 years	Central	notified	0	0	0	25	33	
		rate^b^	0	0	0	217	281	0.004^**^
	Kawempe	notified	153	95	105	120	107	
		rate^b^	239	145	157	176	154	0.0009^***^
	Nakawa	notified	0	0	11	13	37	
		rate^b^	0	0	17	19	55	0.007^**^
	Lubaga	notified	54	56	56	38	34	
		rate^b^	68	49	33	40	19	<0.0001^****^
	Makindye	notified	48	35	24	30	15	
		rate^b^	67	47	32	39	19	<0.0001^****^

^b^notification per 100,000

^*^ *P* ≤ 0.05, ^**^ *P* ≤ 0.01, ^***^ *P* ≤ 0.001, ^****^ *P* ≤ 0.0001

Overall there was a 34% decline in the childhood TB notification rate over the study period with a significant decline observed among the 0 to 4 years’ age group. See the other details in Table 2.

**Table 2:** The trend of paediatric TB notification rates in Kampala 2011–2015

		2011	2012	2013	2014	2015	*p* value for trend
All children	notified	568	417	401	507	433	
	confirmed^b^	49	28	57	112	94	
	population	478,075	487,830	497,785	507,942	518,307	
	rate^a^	119	85	81	100	84	0.07
0–4 years	notified	313	231	209	281	207	
	confirmed^b^	11	4	7	42	8	
	population	200,684	204,780	208,958	213,222	217,573	
	rate^a^	155	112	100	131	95	0.003^**^
5–14 years	notified	255	186	196	226	226	
	confirmed^b^	38	24	50	70	86	
	population	277,390	283,051	288,826	294,720	300,734	
	rate^a^	81	65	67	76	75	0.97

^a^notification per 100,000

^*^ *P* ≤ 0.05, ^**^ *P* ≤ 0.01, ^***^ *P* ≤ 0.001, ^****^ *P* ≤ 0.0001

^b^bacteriologically confirmed either smear, Xpert MTB/RIF or culture

### HIV and ART uptake over the study period

The proportion tested for HIV was 79% (1926/2333) and 100% (1925/1926) received their results of whom 35% (673/1926) were HIV-positive. Of those 91% (610/673) were on ART and 97% (654/673) were on CPT. Details of HIV and ART uptake over the study duration are in Table 3.

**Table 3:** Trend of HIV testing, ART and CPT uptake among children with TB in Kampala 2011–2015

Age group	variable	2011	2012	2013	2014	2015	*P* value for trend
	Notified cases	568	417	401	507	433	
	Tested for HIV	333	264	387	508	433	
Overall	% tested for HIV	59	63	96.5	99.8	100	<0.0001^***^
	Received results	333	264	387	508	433	
	HIV + ve (%)	126 (38)	114 (43)	100 (26)	182 (36)	151 (35)	0.39
	CPT (%)	121 (96)	111 (97)	97 (97)	175 (96)	150 (99)	0.35
	ART (%)	107 (85)	102 (90)	86 (86)	167 (92)	148 (98)	0.003^**^
	Tested for HIV	180	135	197	280	207	
0–4 years	Received results	180	135	197	280	207	
	HIV + ve (%)	57 (32)	53 (39)	37 (19)	98 (35)	62 (30)	0.58
	CPT (%)	55 (96.5)	51 (96.2)	35 (94.6)	94 (95.9)	61 (99)	0.48
	ART (%)	48 (84)	48 (91)	26 (70)	87 (89)	60 (97)	0.58
	Tested for HIV	54	71	81	97	93	
5–9 years	Received results	54	71	81	97	93	
	HIV + ve (%)	36 (67)	41 (58)	26 (32)	38 (39)	42 (45)	0.0001^***^
	CPT (%)	34 (94.4)	40 (97.6)	25 (96.2)	36 (94.7)	42 (100)	0.11
	ART (%)	30 (83)	35 (85)	24 (92)	36 (95)	42 (100)	<0.0001^****^
	Tested for HIV	99	58	109	131	133	
10–14 years	Received results	99	58	109	131	133	
	HIV + ve (%)	33 (33)	20 (34)	37 (34)	46 (35)	47 (35)	0.73
	CPT (%)	32 (97)	20 (100)	37 (100)	45 (97.8)	46 (99)	0.56
	ART (%)	29 (88)	19 (95)	36 (97)	44 (96)	46 (98)	0.0028^**^

^*^ *P* ≤ 0.05, ^**^ *P* ≤ 0.01, ^***^ *P* ≤ 0.001, ^****^ *P* ≤ 0.0001

### Outcomes of notified children TB cases over the study period

The average treatment success rate (TSR) was 77% during the study period, a rate below the national target of 80% and there was no significant increase over the study period. The average TSR was lowest in children 0 to 4 years and adolescents 10 to 14 years at 76% compared with 79% in 5 to 9 years. Overall, we noted the highest decline in loss to follow up in adolescents 10 to 14 years. The details of treatment outcomes during the study period are in Table 4.

**Table 4:** Distribution of the treatment outcomes by age group in children notified with TB in Kampala district 2011–2015

Age group	Outcome	2011 (%)	2012 (%)	2013 (%)	2014 (%)	2015 (%)	*P* value for trend
Overall	^a^Cured	39	50	44	56	77	<0.0001^****^
	^b^TSR	78	70	70	83	83	0.084
	^b^LFU	7	16	5	5	3	0.02^*^
	^b^Died	4	5	10	9	11	0.027^*^
	^b^Not Evaluated	11	9	15	3	3	0.016^*^
0–4 years	^a^Cured	27	25	14	24	63	<0.0001
	^b^TSR	80	71	70	79	82	0.37
	^b^LFU	7	12	4	5	3	0.049^*^
	^b^Died	5	6	10	12	12	0.027^*^
	^b^Not Evaluated	9	11	16	4	2	0.016^*^
5–9 years	^a^Cured	40	60	0	50	80	<0.0001^****^
	^b^TSR	87	66	65	91	84	0.14
	^b^LFU	3	21	10	3	2	0.018^*^
	^b^Died	4	5	8	5	11	0.074
	^b^Not Evaluated	6	8	17	1	3	0.107
10–14 years	^a^Cured	42	53	56	83	77	<0.0001^****^
	^b^TSR	67	72	73	86	84	0.0004^***^
	^b^LFU	10	19	4	7	2	0.0014^**^
	^b^Died	1	4	12	6	9	0.02^*^
	^b^Not Evaluated	22	6	11	2	3	<0.0001^****^

^a^% of PBC cases only; ^b^% of all cases; TSR combines cure and completed cases

^*^ *P* ≤ 0.05, ^**^ *P* ≤ 0.01, ^***^ *P* ≤ 0.001, ^****^ *P* ≤ 0.0001

## Discussion

We undertook to document the trend in notification and outcome of children TB cases in Kampala city. Our findings show a non-significant declining trend of TB notification rate over the study period. The outcomes of TB varied over the study period with no significant increase in the treatment success rate and the average treatment success rate not reaching the national target of 80%. The average mortality rate was highest among the under-fives at 9% almost double the ≤5% WHO acceptable case fatality ratio to achieve the 2025 milestone for reduction of TB deaths [2] .

Our results show a modest declining trend in TB notification rate among children reported in Kampala. We noted that this decrease was not uniform with children under 5 years showing a significant decline in notification rate but a stable trend in the 5 to 14 years. This finding is consistent with what we earlier reported for the 2009 and 2010 [11]. Our results are also comparable to the reported global trend in world TB report 2017 that shows a modest overall decline [2]. We note the higher proportionate TB notification in age group 0 to 4 years over the study period attributable to the increased focus by the national program on TB diagnosis in the under-fives. Since appointing of a paediatric TB focal person at the NTLP, there has been greater impetus in TB case notification among children. A Paediatric TB curriculum was developed and systematic training and mentorships undertaken. A cluster randomized study showed that systematic training increased TB notification in children by three times from the baseline [15]. We note the documented steady decrease of notified TB cases among adults [2]. The decrease in the number of adults with TB represents a lower transmission chance most especially in the home environment [16] and this best reflects in the under-fives that have the highest risk of infection. In this report, we show that children represent about 7% of reported TB in Kampala city but also observe a decrease in the proportion of children over the years though the numbers increased. Compared to the projected estimate of 15% of all TB among children, the current performance remains below the expected [10]. The value addition of this paper above our previous work [11] is demonstration of trend of outcome of TB in children. Our previous work had 2-time points that could not allow us to show trend. This work also covers a period in which several interventions to increase TB case finding in children took place as opposed to of our previous work where minimal took place.

Most of the TB in children (80%) was pulmonary of which the clinically diagnosed represented 82%. This observation might be because of to the extra efforts to train health workers in childhood TB diagnosis and the emphasis on using algorithms to make a clinical diagnosis. Only about 7% of pulmonary bacteriologically confirmed cases had smear microscopy done of whom many (45%) were smear positive. We can speculate this to have resulted from the limited ability of health workers to collect sputum samples. The higher smear positivity rate suggests a selection bias towards older children who are more likely to provide a sputum sample with higher bacillary load. This low sputum collection rate implies the Xpert® MTB/RIF which is the recommended first test for TB has low uptake. We note that even with emphasis on Xpert® MTB/RIF as first test, the bacteriologically confirmed constituted 18% of the PTB cases. This compares with 15% reported in our previous work when the Xpert® MTB/RIF was not readily available [11]. This small difference may suggest underutilization of the Xpert® MTB/RIF in children whose underlying reasons are outside the scope of this work.

The average proportion tested for HIV was still low at 79% over the study period compared to the national target of 100%. There was however a significant increase from 56% in 2011 to 100% in 2015 and all the tested children received their results. This suggests the efforts over the study period in TB and HIV integration were yielding results. The average TB and HIV co-infection was 35% and remained in the same range over the study period with greatest decline seen in the 5–9 years. Data on the prevalence of HIV in children with TB are rare but available literature shows 5 to 56% in different settings [17]. Previous work in our setting reported an HIV prevalence of up to 49% among children with TB [11, 18] therefore our findings show a significant decline in HIV burden among children registered for TB treatment. This could be a reflection of reduced mother to child HIV transmission that has reduced new infections in children from 27,660 in 2011 to 9629 in 2013 [19]. Alternatively, it could be HIV positive children with TB die before diagnosis since they have a higher hospital related mortality [20]. Recent work by Dodd et al. showed the odds of HIV in cohorts of children with TB compared to that of children without TB was 7 times [21]. We found good CPT uptake above 95% and ART uptake increasing from 24% in our previous work [11] to the reported 91%. This is a good sign of integrated TB and HIV care services in accordance to with the Uganda national guidelines.

The average treatment success rate over the study period was 77%. We noted variations in all age groups but 0 to 4 years and 5 to 9 years had declining treatment success rate over the study period. A report from Malawian hospital TB records in 1998 showed poor treatment success rate of less than 43% in children below 5 years and 54% in those above 5 years [22]. In our study, we noted the direct linkage between many cases of loss to follow, high mortality and treatment success rate. We hypothesize this results from the drive to diagnose many more children without satisfactory human resources to support adherence leading to high loss to follow up and poor completion rates. The 10 to 14 years showed the highest loss to follow up in the earlier years of the study period. In most children, diagnosis is made on clinical grounds and where parents do not agree, adherence to treatment remains a challenge. We noted a significant decline in loss to follow up over the study period. The explanation for this significant decline in loss to follow is beyond the scope of this study. Our results show that mortality in the 0 to 4 years age-group increased as the notifications increased. Most of these deaths are likely from the hospital setting where TB diagnosis in children is commonly made late. There is evidence that many children die either as a result or with underlying undiagnosed TB [23]. Since we document a high death rate, we hypothesize that TB in this 0 to 4 years age-group is more likely to be severe and diagnosed late usually at the referral health unit. There is evidence that children with TB present with other conditions most especially severe pneumonia where TB is an afterthought leading to late diagnosis of TB [24, 25]. Harries A et al. reported a higher mortality in the under 1-year and those below 4 years compared to other age groups [22]. A recent systematic review by Jenkins et al. [26] showed a low mortality rate of 0.9% in children with TB in the context of available TB medicines. This may confirm our suggestion that most of deaths are due to late diagnosis of TB rather than failure of TB medicines. The Adolescent age group had a high average mortality rate of about 6%. This high mortality in adolescents has largely gone unnoticed whose causes our study could not find out. The adolescents commonly have adult type disease and are less likely to be compliant to chronic treatment [27]. The poor TB treatment outcomes among children needs further exploration and there is need to examine the time points of these deaths on the path of care and other co-morbidities in the adolescents.

### Strengths and limitations

In this paper, we showed a trend over a five-year study period. This report follows a previous study with similar methods and therefore allows for a fair assessment of the progress of childhood TB services. We admit the inherent weaknesses of retrospective data that includes missing and inability to verify the records. Our inability to report data on Xpert ® MTB/RIF use in the diagnosis of TB in children is a drawback. The routine reporting tools to the NTLP were not capturing this data during the period covered by this study. We are however confident that our results are a representation of the true picture of the trend of childhood TB notification and outcomes in Kampala city. The weakness of notification rate is that it does not reflect the prevailing TB incidence unless there are few numbers of undetected, unreported or retreatment cases alongside good access to health services. Besides, the denominator projected population assumed a uniform growth rate despite the migrations in the urban setting, limiting accurate measurement of TB notification rates. We are also aware that some of the patients registered in Kampala are nonresidents and that some registered in one division may be residing in another division. Nevertheless, for purposes of showing a trend, our paper still makes an important contribution.

### Implications for practice and program implementation

The focused efforts by the NTLP to look for TB among the under 15 years age group did show good results. The declining trend of notification rates in children mirrored declining adult TB notifications. This study cannot explain drivers of the noted trend for which we recommend further study. The low proportion of children among the notified TB cases in Kampala is likely due to a weak case finding system. Weak household contact tracing, TB screening and diagnostic skills lead to under diagnosis and under reporting TB in children. The high loss to follow up may suggest weaknesses in the support services such as adherence counseling and human resources for health leading to inadequate quality of care. We hypothesize the high mortality rate is mainly among previously hospitalized children. There is high mortality of up to 11% among children diagnosed with TB after hospitalization with delays of up to 16 days to make a TB diagnosis [28]. Thus, many children started on TB treatment while on the wards are commonly sicker.

## Conclusion

There was a minimal decline in the trend of childhood TB notification rates from our report. The increase in the treatment success rate over the study period was not significant and its average did not reach the national target. The adolescents had the worst proportionate treatment outcomes. The average mortality over the study period of about 5% is still high with under-fives having a consistently higher relative mortality.

## Abbreviations

ART: Anti-retroviral therapy
CPT: Cotrimoxazole preventive therapy
DTU: Diagnostic and treatment unit
EPTB: Extra-pulmonary tuberculosis
HIV: Human immune-deficiency virus
MDG: Millenium development goal
MoH: Ministry of Health
MREC: Mulago Research and Ethics Committee
NTLP: National Tuberculosis and Leprosy Program
PTB: Pulmonary tuberculosis
TB: Tuberculosis
TSR: Treatment success rate
WHO: World Health Organization
